# Supplementary material for: Live Cell Imaging of Bioorthogonally Labelled Proteins Generated With a Single Pyrrolysine tRNA Gene
Source: Sci Rep. 2018 Sep 28;8:14527. doi: 10.1038/s41598-018-32824-1 (PMC6162220; doi:10.1038/s41598-018-32824-1)
Supplement: Supplementary file 1 — Supplementary Information [file 41598_2018_32824_MOESM1_ESM.pdf]

Supplementary Information

Live Cell Imaging of Bioorthogonally Labelled Proteins Generated With a Single Pyrrolysine tRNA Gene

Noa Aloush[a,c], Tomer Schvartz[b,c], Andres I. König[b,c], Sarit Cohen[a,c], Eugene Brozgol[d], Benjamin Tam[a], Dikla Nachmias[b,c], Oshrit Ben-David[a,c], Yuval Garini[d], Natalie Elia[b,c], and Eyal Arbely *[a,b,c]

[a]Department of Chemistry, Ben Gurion University of the Negev, P.O. Box 653, Beer-Sheva, 8410501, Israel

[b]Department of Life Sciences, Ben Gurion University of the Negev, P.O. Box 653, Beer-Sheva, 8410501, Israel

[c]National Institute for Biotechnology in the Negev, Ben-Gurion University of the Negev, P.O. Box 653, Beer-Sheva, 8410501, Israel

[d]Physics Department and Institute for Nanotechnology, Bar Ilan University, Ramat Gan, 5290002, Israel

*Corresponding Author: arbely@bgu.ac.il

Supplementary Text

General introduction

Genetic code expansion technology enables the genetic encoding and site specific incorporation of NCAAs into ribosomally synthesized proteins. Currently used methods usually rely on the amber stop codon, TAG, and on an orthogonal pair of amber suppressor $tRNA_{CUA}$ and aminoacyl-tRNA synthetase. A frequently used pair is the pyrrolysyl-tRNA synthetase/$tRNA_{CUA}$ pair from *Methanosarcina* species, which is inert (orthogonal) to synthetases and tRNAs in *E. coli*, yeast, mammalian cells, and multicellular organisms. In a typical study, an in-frame TAG mutation is introduced at a specific position within the gene of interest, which is then co-expressed with the orthogonal synthetase/$tRNA_{CUA}$ pair. In some cases, the wild type aaRS can recognize the NCAA, but in most cases the aaRS is evolved (by means of directed evolution) to recognize the NCAA and not one of the canonical amino acids. A functional aaRS can aminoacylate its cognate tRNA with the NCAA and the charged tRNA enables the co-translational incorporation of the NCAA in response to the in-frame UAG codon. The result, is a full-length protein with a site-specifically incorporated NCAA that was encoded by an in frame TAG stop codon. The advantage of site-specific incorporation of NCAAs is that it facilitates the study of homogeneously modified full-length proteins both in vitro and in cultured mammalian cells.

The attachment of a fluorescent dye to a specific protein in a cell, requires the formation of a covalent bond between two unique chemical groups; one installed on a surface-exposed residue, and the other attached to the fluorescent dye. While the chemical synthesis of modified fluorescent dyes is usually feasible, the addition of a unique chemical group to proteins in live cells became possible only recently, using genetic code expansion technology. Through this approach the site-specifically incorporated NCAA carries a unique chemical handle that can form a covalent bond with the modified fluorescent dye within the complex chemical environment of the cells in a non-invasive and non-toxic manner.

Chemical reactions that enable the formation of a specific chemical bond within the cellular environment are often referred to as bioorthogonal reactions. Under this category, one of the fastest reactions is the inverse electron-demand Diels-Alder reaction between strained alkenes or alkynes and tetrazines. These two chemical groups are relatively stable within the cellular environment and react to form a covalent bond with nitrogen as the only byproduct. Importantly, the fluorescence of the fluorophore is quenched when it is conjugated to the unbound tetrazine, but fluorescence increases upon reaction of the tetrazine moiety with the strained alkyne or alkene

(i.e., a fluorogenic reaction). The quenching properties of unbound tetrazine-fluorophores can significantly reduce background fluorescence originating from residual unbound chromophores, thereby improving signal-to-noise ratio. Maintaining high signal-to-noise ratio is critical for the performance of both high-speed single-molecule tracking and for super resolution imaging.

Supplementary Tables

Table S1. Selected studies describing genetic code expansion in mammalian cells.

Paper	Orthogonal tRNA	Orthogonal synthetase	No. of plasmids	tRNA promoter	No. of tRNA genes	Cell lines	tRNA copy number evaluated	Transfection method
Buvoli et al. 2000[1]	Human Ser	Human Ser	2	Native promoter with different spacers	1, 8, 16	COS7	Yes	Transient
Sakamoto et al. 2002[2]	*Bacillus stear.* Tyr *E. coli* Tyr	*E. coli* Tyr	2	5'-flanking region human Tyr tRNA	1, 9	CHO	Yes	Stable, transient
Hino et al. 2005[3]	*Bacillus stear.* Tyr	*E. coli* Tyr	2	5'-flanking region human Tyr tRNA	9	CHO	No	Transient
Hino et al. 2007[4]	*Bacillus stear.* Tyr	*E. coli* Tyr	3	5'-flanking region human Tyr tRNA	9	CHO, HEK293	No	Transient
Wang et al. 2007[5]	*E. coli* Tyr	*E. coli* Tyr	2	H1	1	HeLa, HEK293, HEK293T, mouse hippocampal neurons	No	Stable, transient

**Paper**	**Orthogonal tRNA**	**Orthogonal synthetase**	**No. of plasmids**	**tRNA promoter**	**No. of tRNA genes**	**Cell lines**	**tRNA copy number evaluated**	**Transfection method**
Liu et al. 2007[6]	*Bacillus stear.* Tyr	*E. coli* Tyr	2 or 3	5'-flanking region human Tyr tRNA	3	CHO, HEK293	Indirectly	Transient
Mukai et al. 2008[7]	*M. mazei* Pyl	*M. mazei* Pyl	3	U6(9×tRNA), CMV+U6 or T7 (1×tRNA)	1, 9	CHO, HEK293 c-18	Yes	Transient
Chen et al. 2009[8]	*M. mazei* Pyl	*M. mazei* Pyl	2	U6	1	CHO, HEK293	No	Transient
Gautier et al. 2010[9]	*M. mazei* Pyl	*M. mazei* Pyl	2	CMV-enhancer+U6	4	HEK293	No	Transient
Hayashiet al. 2011[10]	*E. coli* Tyr	*E. coli* Tyr		5'-flanking region human Tyr tRNA	3	CHO	No	Transient
Coin et al. 2011[11]	*E. coli* Tyr	*E. coli* Tyr	1	H1	1, 2, 3, 4	HEK293	Yes	Transient
Shen et al. 2011[12]	*E. coli* Tyr	*E. coli* Tyr	2 or 3 viruses	H1	4	HeLa, HEK293, HCN-A94	No	Lentiviral
Coin et al. 2013[13]	*Bacillus stear.* Tyr	*E. coli* Tyr	2	U6	3	HEK293	No	Transient
Kang et al. 2013[14]	*E. coli* Leu	*E. coli* Leu	2	H1	1, 3	HEK293, primary neurons, in vivo	No	Transient

**Paper**	**Orthogonal tRNA**	**Orthogonal synthetase**	**No. of plasmids**	**tRNA promoter**	**No. of tRNA genes**	**Cell lines**	**tRNA copy number evaluated**	**Transfection method**
Chatterjee et al. 2013[15]	*M. mazei* Pyl, *D. hafnie.* Pyl, *E. coli* Tyr, *Bacillus stear.* Tyr	*M. barkery* Pyl *E. coli* Tyr	1 or 2 viruses	H1, U6	4	HEK293, 3T3, MEF, RCF, HeLa	No	Baculovirus
Xiao et al. 2013[16]	*M. mazei* Pyl, *D. hafnie.* Pyl, *E. coli* Tyr, *Bacillus stear.* Tyr	*M. barkery* Pyl *E. coli* Tyr		H1, U6	4	HEK293, Freestyle 293-F	No	Transient
Schmied et al. 2014[17]	*M. mazei* Pyl	*M. mazei* Pyl	2	U6, CMV-enhancer+U6	1, 4, 8	HEK293	Yes	Transient
Uttamapinant et al. 2015[18]	*M. mazei* Pyl *M. barkery* Pyl	*M. mazei* Pyl	2	U6, CMV-enhancer+U6	4, 8	HEK293, COS7	Yes	Transient
Elsässer et al. 2016[19]	*M. mazei* Pyl	*M. mazei* Pyl	2	U6	8	HEK293, E14 mouse ESC, HCT116, MEF, 3T3	No	Stable (Piggy-Bac)
Zheng et al. 2016[20]	*E. coli* Tyr, *Bacillus stear.* Tyr	*E. coli* Tyr	1, 2, or 3 viruses	H1, U6	Variable	HEK293, ex vivo mouse brain slice culture	Yes	Baculovirus

Paper	Orthogonal tRNA	Orthogonal synthetase	No. of plasmids	tRNA promoter	No. of tRNA genes	Cell lines	tRNA copy number evaluated	Transfection method
Peng et al. 2016[21]	*M. mazei* Pyl	*M. mazei* Pyl	2	U6	4	HEK293, HeLa	No	Transient
Serflin et al. 2017[22]	*M. mazei* Pyl	*M. barkery* Pyl	2	U6	1, 4	HEK293	Yes	Transient

Supplementary Figures

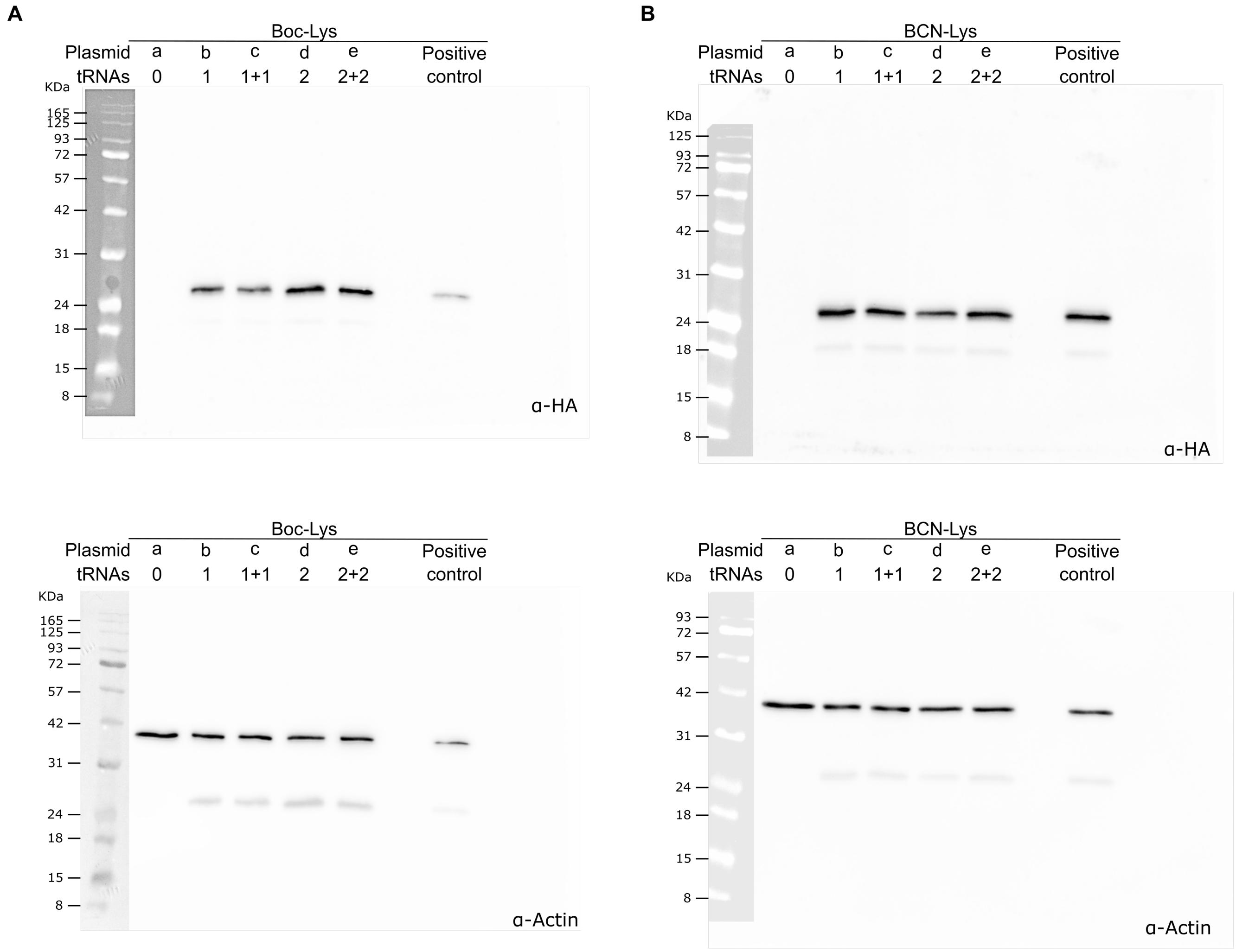

Figure S1. Full-length Western blots displayed in Fig. 2A. COS7 cells were transfected with plasmids **a**–**e** carrying either Pyl-RS for the incorporation of **1** (**A**), or evolved BCN-RS for the incorporation of **2** (**B**). Top panel: Western blotting using anti-HA primary antibody. Bottom panel: Western blotting using anti actin primary antibody.

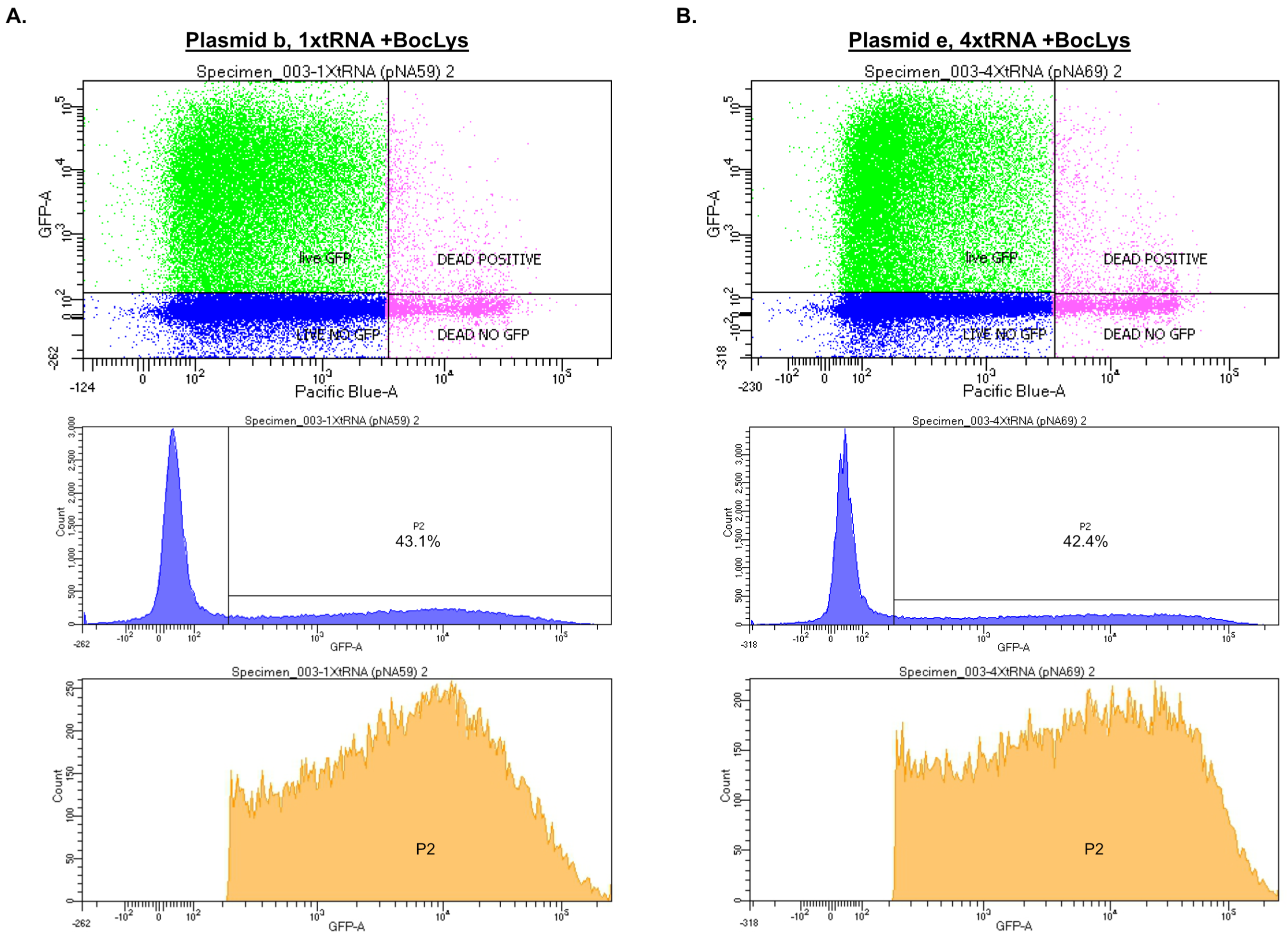

Figure S2. FACS analysis of COS7 cells transfected with plasmid **b** or plasmid **e** (panel **A** or **B**, respectively) and incubated with ncAA **1**. See also Figure 2B.

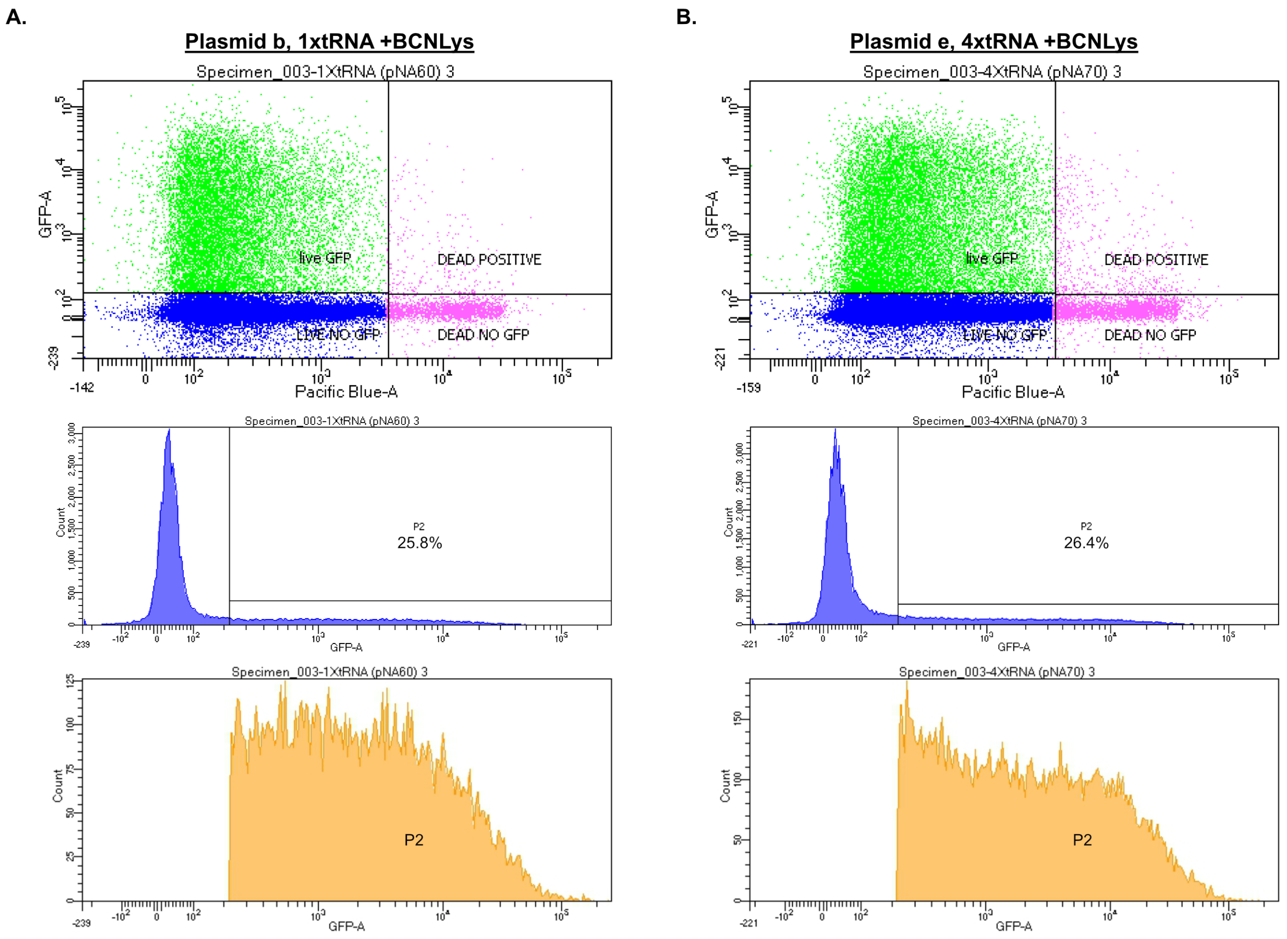

Figure S3. FACS analysis of COS7 cells transfected with plasmid **b** or plasmid **e** (panel **A** or **B**, respectively) and incubated with ncAA **2**. See also Figure 2B.

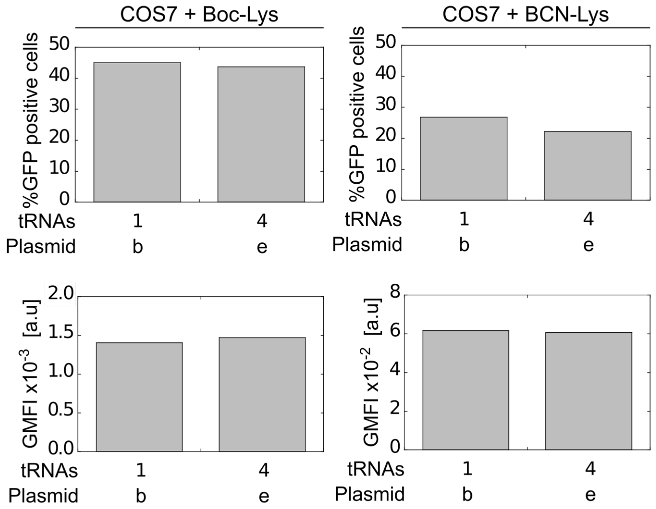

Figure S4. Percentage of GFP-positive cells and GFP expression levels measured 24 h post-transfection. COS7 cells were transiently transfected with plasmids carrying either Pyl-RS or BCN-RS and 1 or 4 copies of PylT. Transfected cells incubated with indicated NCAA for 24 h and EGFP expression was analysed by FACS. Overall efficiency was calculated as percentage of GFP-positive cells. Protein expression level was calculated from GFP fluorescence intensity (GFP-area) and displayed as geometric mean fluorescence intensity (GMFI).

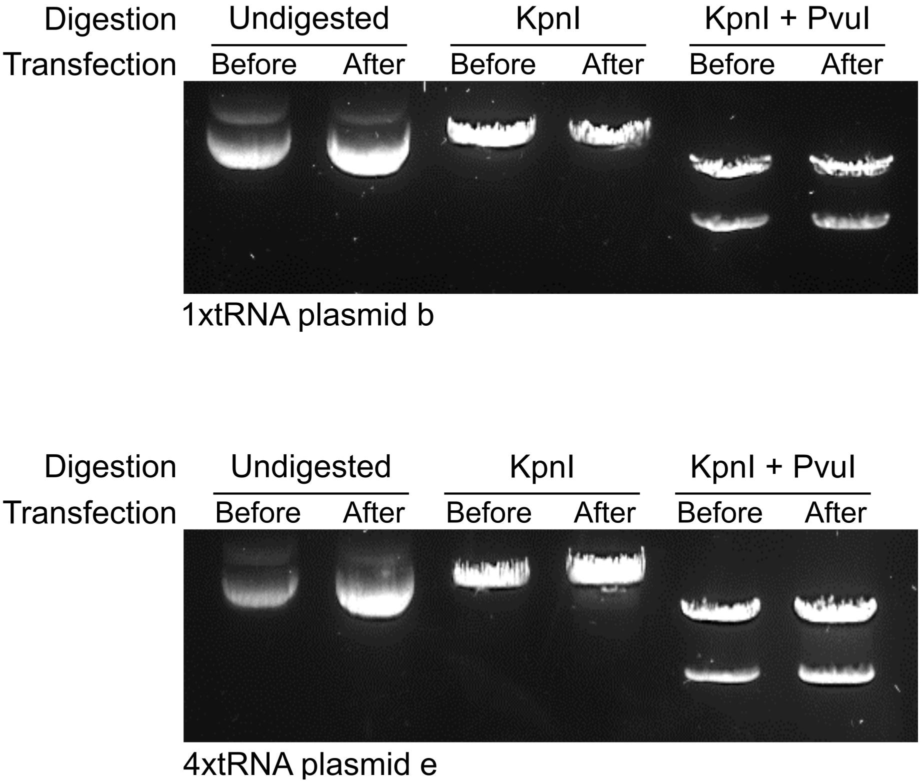

Figure S5. Plasmid stability in transiently transfected cells. COS7 cells were transfected in the presence of NCAA **1** with 1×PylT plasmid **b** or 2+2×PylT plasmid **e**. Plasmids rescued 48 hr post transfection and the size of rescued plasmid was analysed by agarose-gel electrophoresis. To better compare between pre- and post-transfected plasmids, circular plasmids were compared to plasmids digested with KpnI, or both KpnI and PvuI.

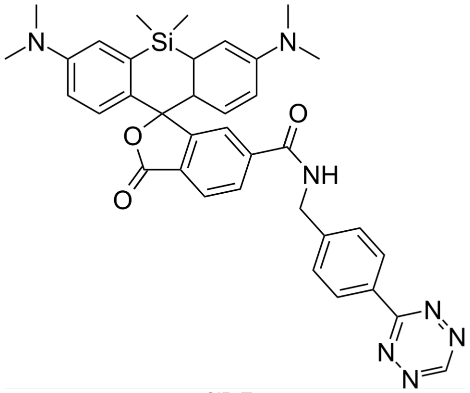

Figure S6. Chemical structure of 4-(1,2,4,5-tetrazine-3-yl)benzylamino conjugated silicon rhodamine (SiR-Tet).

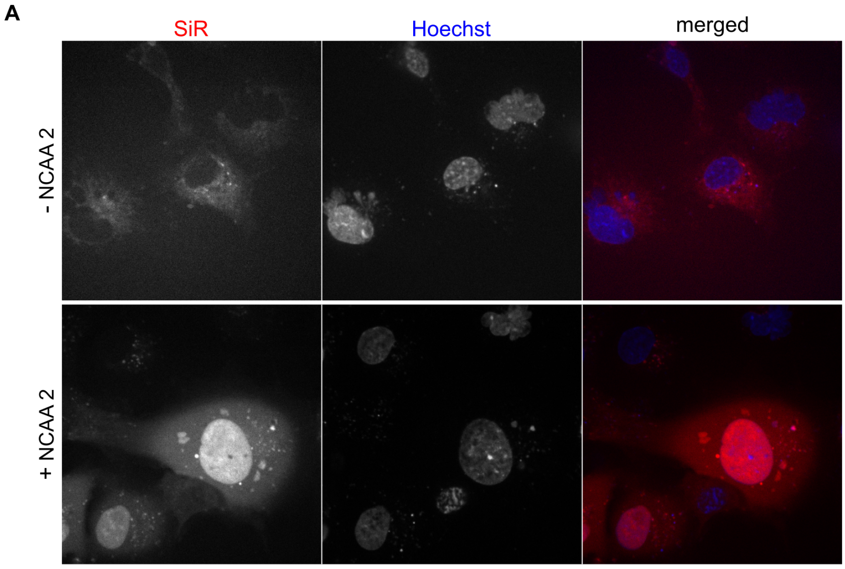

Figure S7. Background SiR-Tet labelling in COS7 cells. Cells were transiently transfected with 4×PylT plasmid **k** carrying BCN-RS and incubated in the absence (top row) or presence (bottom row) of NCAA **2**. 48 h post-transfection the cells were incubated with SiR-Tet. Compared to transfected COS7 cells incubated in the presence of NCAA **2**, low background fluorescence was observed in the cytosol and nucleus of cells incubated in the absence of NCAA **2**, suggesting that background labelling is NCAA-dependent.

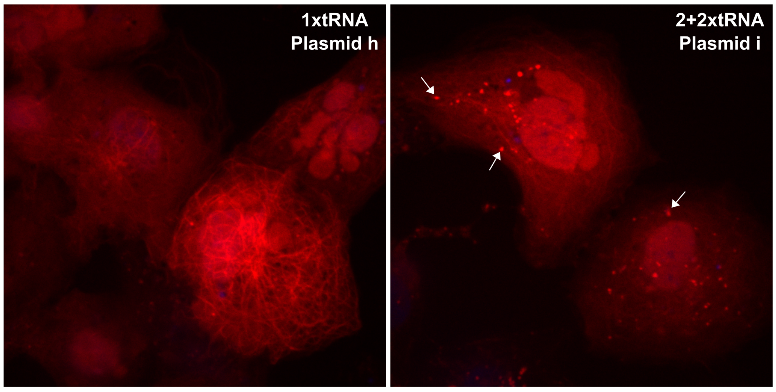

Figure S8. Live cell imaging of SiR-labelled 45BCNK-α-tubulin co-expressed with 1×PylT plasmid (left) or 2+2×PylT (right), in COS7 cells . White arrows mark dense ‘fluorescent dots’ often observed in cells transfected with 2+2×PylT plasmids.

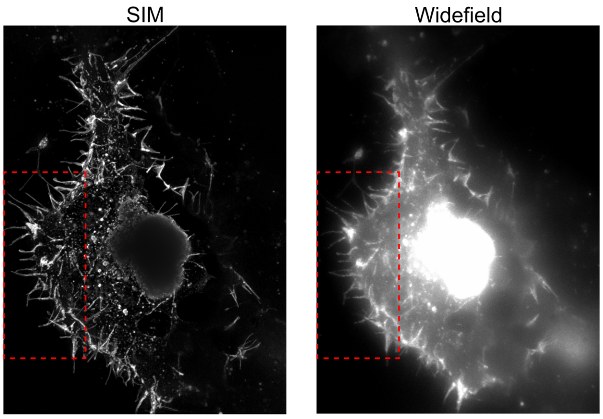

Figure S9. SIM imaging of SiR-labelled 150BCNK-EGFP-CAAX. Red rectangles mark the zoomed-in regions presented in Figure 6.

Supplementary Methods

General

General chemicals were purchased from Sigma-Aldrich or Fisher scientific. Boc-Lys was purchased from Chemimpex (Wood Dale, IL), BCN-Lys from Synaffix (Oss, Netherlands) and Zeocin from InvivoGen (San Diego, California). *E. coli* DH10B strain (Life Technologies) was used for cloning and plasmid propagation and grown at 37°C in liquid LB medium or on LB-agar plates supplemented with Zeocin (35 µgr/mL in liquid medium, 25 µgr/mL in LB-agar plates). Media components were obtained from BD-Difco (Franklin Lakes, NJ). PCR was performed using Phusion High-Fidelity DNA polymerase (NEB) according to the manufacturer's protocol. Restriction endonucleases and T4 DNA ligase were purchased from New England Biolabs and used according to the manufacturer's protocols. Plasmid DNA was purified using NucleoSpin Plasmid kit (Macherey-Nagel, Bethlehem, PA) and PCR products were generally purified from agarose gel using NucleoSpin Gel and PCR Clean-up kit (Macherey-Nagel, Bethlehem, PA). All oligonucleotides for PCR were purchased from Sigma-Aldrich and DNA sequencing was performed in-house at the DNA Microarray and Sequencing Unit (Ben-Gurion University). De-novo synthesized genes were purchased from IDT (Israel). COS7 and HEK293T cells were cultured in DMEM (Biological Industries, Israel) supplemented with 10% heat inactivated fetal bovine serum, penicillin (100 Units/mL), and streptomycin (0.3 mg/mL, all supplements were purchased from Biological Industries, Israel). SiR-Tet was purchased from Spirochrome (Switzerland).

Plasmid Construction

All plasmids used in the course of this study were based on the backbone of commercially available pBudCE4.1 vector (Invitrogen). Wild-type *methanosarcina mazei* pyrrolysine synthetase, BCN-RS (Y306M, L309G, and C348A, relative to wild-type synthetase), as well as 1× or 2×U6-(U25C)PylT cassettes were synthesized de novo by IDT. The expression vectors were cloned in three steps. First, wild type Pyl-RS or BCN-RS were cloned into pBudCE4.1 between BamHI and EcoRI sites, downstream of the CMV promoter. Second, a tRNA cassettes was cloned using the single NheI restriction site (for vectors with 1 or 2 copies of PylT) and if required, a second tRNA cassette was cloned using the single PvuI restriction site (for vectors with 1+1 or 2+2 copies of PylT). This cloning step provided two sets of plasmids; one set for the expression of wild type Pyl-RS and the other set for the expression of BCN-RS, where each set is composed

of five plasmids harboring indicated synthetase and 0, 1, 1+1, 2, or 2+2 copies of PylT genes. In the third cloning step the gene of interest was cloned into the ten plasmids generated in the second cloning step, between KpnI and XhoI sites, downstream of the EF1α promoter.

An in-frame TAG mutation in position 150 of EGFP (pEGFP-N1 vector, Clonetech) was introduced by site directed mutagenesis. $EGFP^{150TAG}$-HA, was amplified by PCR and cloned into above described plasmids, using KpnI and XhoI sites. This cloning step generated a series of 10 plasmids for the expression of $EGFP^{150TAG}$-HA (controlled by EF1α promoter) together with either Pyl-RS or BCN-RS (controlled by CMV promoter), each plasmid harboring the indicated copy-number of *PylT*. HA-α-Tubulin with an in-frame TAG mutation at position G45 was cloned in the same way to generate 1× or 2+2×PylT plasmids harboring HA-α-$tubulin^{45TAG}$ and BCN-RS. Amber stop codon mutation in positions N150, was introduced into EGFP with C-terminal CAAX motif by site-directed mutagenesis. The $EGFP^{150TAG}$-CAAX gene was digested using NotI and KpnI, and cloned into above described plasmids, harboring 1× or 2+2×PylT and BCN-RS.

Cell culture

Cells were cultured at 37°C in a humidified chamber with 5% CO_2 and seeded as follows. For Western blot and FACS analyses: Twenty-four hours before transfection, 100,000 cells were seeded per well, in a 24-well plate. For Live cells imaging: Twenty-four hours before transfection, 30,000 cells were seeded per well, in μ-Slide 4 Well Glass Bottom plate (ibidi, Martinsried, Germany) for live-cell imaging, or in μ-Dish 35 mm, high Glass Bottom (ibidi, Martinsried, Germany) for imaging of fixed cells. Half an hour before transfection, the media was changed to fresh growth media containing BOC-Lys (1 mM) or BCN-Lys (0.5 mM). Transient transfection was performed using Lipofectamine 2000 (Invitrogen) at reagent:DNA ratio of 5:1, following manufacturer's protocol.

Western blotting

COS7 cells and HEK293T cells were cultured in 24-well plates, transfected as describe above, and incubated with 1 mM Boc-Lys or 0.5 mM BCN-Lys for 48 hours. Cells were lysed in 75 μl RIPA buffer (50 mM Tris pH=8.0, 150 mM NaCl, 1% Triton, 0.5% Sodium deoxycholate and 0.1% SDS) supplemented with protease inhibitors (1.2 mg/ml Leupeptin, 1 mM Pepstatin A, 100 mM PMSF and 1 mg/ml Aprotinin). Protein concentrations were measured by BCA assay kit (ThermoFisher Scientific) and samples were normalized accordingly. Lysates were denatured at

95°C for 5 minutes, and separated by sodium dodecyl sulfate polyacrylamide gel electrophoresis (SDS-PAGE). Proteins were transferred to 0.2 μm nitrocellulose membrane using semi-dry transfer apparatus (Bio Rad, Trans-Blot Turbo). The membrane was blocked for 1 hr with Tris-buffered saline containing 0.05% Tween-20 (TBST) and 5 w/v% non-fat dry milk, followed by an overnight incubation with primary antibody diluted in 5 w/v% bovine serum albumin (BSA, Sigma Aldrich, Israel) in TBST, at 4°C. The membrane was washed with TBST and incubated for 1 h with horse radish peroxidase-conjugated secondary antibody (diluted in 5 w/v% non-fat dry milk in TBST) at room temperature. The membrane was washed again with TBST and developed using ECL reagent (GE Healthcare). The following antibodies were used at the indicated dilutions: mouse-anti-HA-tag (Abm) 1:1000 goat-anti-mouse horseradish peroxidase conjugated secondary antibody (Abcam) 1:10000, and mouse-anti-actin (Abm) 1:20000. Gel bands were quantified using ImageJ.

Plasmid rescue experiments

COS7 cells were cultured in 10 cm plates, transfected as describe above and incubated with 1 mM Boc-Lys for forty-eight hours. Cells were then washed with fresh DMEM and detached using Trypsin (0.25%)-EDTA (0.05%) solution (biological industries, Israel) at 37°C in 5% CO_2 for 5 minutes. Trypsin-EDTA mixture was neutralized with ice cold full-DMEM and cells were centrifuged at 500 g for 5 minutes. Cells were washed three times with ice-cold PBS, and plasmid DNA was purified using NucleoSpin Plasmid kit. Purified DNA was then transformed into competent *E. coli* DH10B cells, and transformed bacteria were incubated over night in LB-media supplemented with 25 µgr/mL Zeocin. Plasmid DNA was purified again using NucleoSpin Plasmid kit and analyzed by agarose gel electrophoresis.

FACS analysis

COS7 cells were cultured in 24-well plates, transfected as describe above and incubated with 1 mM Boc-Lys or 0.5 mM BCN-Lys for twenty-four or forty-eight hours. Cells were detached using Trypsin (0.25%)-EDTA (0.05%) solution (biological industries, Israel) at 37°C in 5% CO_2 for 5 minutes. Trypsin-EDTA mixture was neutralized with ice cold full-DMEM and cells were centrifuged at 500 g for 5 minutes. Cells were washed once with ice-cold 10 mM EDTA in phosphate-buffered saline (PBS), and once with ice-cold 10 mM EDTA and 1 mg/mL DAPI (Life Technologies) in PBS, transferred to FACS tubes (Falcon) and kept at 4°C until analysis. FACS analysis was performed using a BD FACSAria cell sorter.

Confocal fluorescence imaging

Live-cell imaging: COS7 cells were cultured in μ-Slide 4 well glass bottom dish (ibidi). Cells were transfected as describe above and 4 h post transfection, the media was changed to fresh growth media containing 0.5 mM BCN-Lys and ascorbic acid at a final concentration of 100 μM. 48 h post transfection, cells were washed 6 times with fresh full-DMEM over 2 hours. Cells were labelled for 1 h at room temperature with SiR-tet at a final concentration of 600 nM in full-DMEM without FBS. Thereafter, cells were washed 6 times with fresh full-DMEM over 2 h and imaged.

Imaging of fixed cells: COS7 cells were cultured in 35 mm high glass bottom μ-Dish (ibidi) treated as described above for live-cell imaging, washed once with cytoskeleton buffer (Sigma Aldrich, Israel), fixed with 2 mM ethylene glycol bis[succinimidyl succinate] in cytoskeleton buffer for 10 min at 37°C, and permeabilized with 0.2% NP40 in cytoskeleton buffer for 1 min at room temperature. Cells were washed with PBS, 100 mM glycine in PBS, and PBS, before imaging.

Three-dimensional Z-stacks of selected SiR-labelled COS7 cells were collected using a fully incubated confocal spinning-disk microscope (Marianas; Intelligent Imaging, Denver, CO) with a 63×oil objective (numerical aperture, 1.4). Images were recorded on an electron-multiplying charge-coupled device camera (pixel size 0.079 µm; Evolve; Photometrics, Tucson, AZ). Image processing and analysis were done using SlideBook version 6 (Intelligent Imaging).

FLIP experiments: Single Z slices were imaged with a 40×oil objective (NA, 1.3) at 1 s intervals for a total of 2 minutes. A region of interest (ROI) in the cytosol was repetitively photobleached with a 405 nm laser (every other frame for 36 repetitions). The exact same imaging and bleaching parameters were applied in all experiments and conditions. For analysis, the mean intensity of the bleached ROI and of ROIs in the cytosol and nucleus, as well as in the cytosol of a neighboring cell, were plotted overtime.

Confocal microscopy using a single molecule setup: Cells were placed in 37°C incubator (LCI) with 5% CO_2 level on a Single Molecule Detection Platform (Leica TCS SP8 SMD). Imaging was performed with a Leica HC PL APO 63×/1.20 W objective, using HeNe (633 nm) laser. Signal was detected by HyD SMD detector within the detection window (650–800 nm). Four times line averaging was used during acquisition.

Structured illumination microscopy

Three-dimensional Z-stacks of selected SiR-labelled COS7 cells were collected (with sections of 0.144 μm) in three rotations using an ELYRA PS.1 microscope (Carl Zeiss, Jena, Germany). Images were reconstructed using ZEN software (Carl Zeiss) based on the structured illumination algorithm developed by Heintzmann and Cremer.[23] All measurements were performed on reconstructed super resolution images using ZEN.

Supplementary References

[1] Buvoli, M., Buvoli, A. & Leinwand, L. a. Suppression of Nonsense Mutations in Cell Culture and Mice by Multimerized Suppressor tRNA Genes. *Molecular and Cellular Biology* **20**, 3116–3124 (2000).

[2] Sakamoto, K. *et al.* Site-specific incorporation of an unnatural amino acid into proteins in mammalian cells. *Nucleic Acids Research* **30**, 4692–9 (2002).

[3] Hino, N. *et al.* Protein photo-cross-linking in mammalian cells by site-specific incorporation of a photoreactive amino acid. *Nature Methods* **2**, 201–206 (2005).

[4] Hino, N., Hayashi, A., Sakamoto, K. & Yokoyama, S. Site-specific incorporation of non-natural amino acids into proteins in mammalian cells with an expanded genetic code. *Nature Protocols* **1**, 2957–2962 (2007).

[5] Wang, W. *et al.* Genetically encoding unnatural amino acids for cellular and neuronal studies. *Nature Neuroscience* **10**, 1063–1072 (2007).

[6] Liu, W., Brock, A., Chen, S., Chen, S. & Schultz, P. G. Genetic incorporation of unnatural amino acids into proteins in mammalian cells. *Nature Methods* **4**, 239–244 (2007).

[7] Mukai, T. *et al.* Adding l-lysine derivatives to the genetic code of mammalian cells with engineered pyrrolysyl-tRNA synthetases. *Biochemical and Biophysical Research Communications* **371**, 818–822 (2008).

[8] Chen, P. R. *et al.* A facile system for encoding unnatural amino acids in mammalian cells. *Angewandte Chemie (International ed. in English)* **48**, 4052–5 (2009).

[9] Gautier, A. *et al.* Genetically Encoded Photocontrol of Protein Localization in Mammalian Cells. *Journal of the American Chemical Society* **132**, 4086–4088 (2010).

[10] Hayashi, A. *et al.* Dissecting Cell Signaling Pathways with Genetically Encoded 3-Iodo-L-tyrosine. *ChemBioChem* **12**, 387–389 (2011).

[11] Coin, I., Perrin, M. H., Vale, W. W. & Wang, L. Photo-Cross-Linkers Incorporated into G-Protein-Coupled Receptors in Mammalian Cells: A Ligand Comparison. *Angewandte Chemie International Edition* **50**, 8077–8081 (2011).

[12] Shen, B. *et al.* Genetically Encoding Unnatural Amino Acids in Neural Stem Cells and Optically Reporting Voltage-Sensitive Domain Changes in Differentiated Neurons. *Stem Cells* **29**, 1231–1240 (2011).

[13] Coin, I. *et al.* Genetically Encoded Chemical Probes in Cells Reveal the Binding Path of Urocortin-I to CRF Class B GPCR. *Cell* **155**, 1258–1269 (2013).

[14] Kang, J.-Y. *et al.* In Vivo Expression of a Light-Activatable Potassium Channel Using Unnatural Amino Acids. *Neuron* **80**, 358–370 (2013).

[15] Chatterjee, A., Xiao, H., Bollong, M., Ai, H.-W. & Schultz, P. G. Efficient viral delivery system

for unnatural amino acid mutagenesis in mammalian cells. *Proceedings of the National Academy of Sciences* **110**, 11803–11808 (2013).

[16] Xiao, H. *et al.* Genetic Incorporation of Multiple Unnatural Amino Acids into Proteins in Mammalian Cells. *Angewandte Chemie International Edition* **52**, 14080–14083 (2013).

[17] Schmied, W. H., Elsässer, S. J., Uttamapinant, C. & Chin, J. W. Efficient Multisite Unnatural Amino Acid Incorporation in Mammalian Cells via Optimized Pyrrolysyl tRNA Synthetase/tRNA Expression and Engineered eRF1. *Journal of the American Chemical Society* **136**, 15577–15583 (2014).

[18] Uttamapinant, C. *et al.* Genetic Code Expansion Enables Live-Cell and Super-Resolution Imaging of Site-Specifically Labeled Cellular Proteins. *Journal of the American Chemical Society* **137**, 4602–4605 (2015).

[19] Elsässer, S. J., Ernst, R. J., Walker, O. S. & Chin, J. W. Genetic code expansion in stable cell lines enables encoded chromatin modification. *Nature Methods* **13**, 158–164 (2016).

[20] Zheng, Y., Lewis, T. L., Igo, P., Polleux, F. & Chatterjee, A. Virus-Enabled Optimization and Delivery of the Genetic Machinery for Efficient Unnatural Amino Acid Mutagenesis in Mammalian Cells and Tissues. *ACS Synthetic Biology* **6**, 13–18 (2017).

[21] Peng, T. & Hang, H. C. Site-Specific Bioorthogonal Labeling for Fluorescence Imaging of Intracellular Proteins in Living Cells. *Journal of the American Chemical Society* **138**, 14423–14433 (2016).

[22] Serfling, R. *et al.* Designer tRNAs for efficient incorporation of non-canonical amino acids by the pyrrolysine system in mammalian cells. *Nucleic Acids Research* (2017).

[23] Heintzmann, R. & Cremer, C. G. Laterally modulated excitation microscopy: improvement of resolution by using a diffraction grating. *Proc. SPIE 3568, Optical Biopsies and Microscopic Techniques III* **3568**, 185–196 (1999).
